# Numerical Simulation of Fatigue Life of Rubber Concrete on the Mesoscale

**DOI:** 10.3390/polym15092048

**Published:** 2023-04-25

**Authors:** Xianfeng Pei, Xiaoyu Huang, Houmin Li, Zhou Cao, Zijiang Yang, Dingyi Hao, Kai Min, Wenchao Li, Cai Liu, Shuai Wang, Keyang Wu

**Affiliations:** 1 School of Engineering, Architecture and The Environment, Hubei University of Technology, Wuhan 430068, China; 102100805@hbut.edu.cn (X.P.); huangxiaoyu202203@163.com (X.H.); a424185749@163.com (D.H.); c1357962470@163.com (K.M.); csceczj319025@163.com (W.L.); zjsjygszh2023@163.com (C.L.); 2 China Construction Third Bureau First Engineering Co., Ltd., Wuhan 430040, China; haodingyi1015@163.com (Z.C.); mk294588366@163.com (Z.Y.); 3 Wuhan Construction Engineering Co., Ltd., Wuhan 430056, China; wangshuai@wceg.com.cn (S.W.); wukeyang@wceg.com.cn (K.W.)

**Keywords:** numerical simulation, rubber concrete, fatigue life, three-point bending, polymer, mesoscale model

## Abstract

Rubber concrete (RC) exhibits high durability due to the rubber admixture. It is widely used in a large number of fatigue-resistant structures. Mesoscale studies are used to study the composition of polymers, but there is no method for fatigue simulation of RC. Therefore, this paper presents a finite element modeling approach to study the fatigue problem of RC on the mesoscale, which includes the random generation of the main components of the RC mesoscale structure. We also model the interfacial transition zone (ITZ) of aggregate mortar and the ITZ of rubber mortar. This paper combines the theory of concrete damage to plastic with the method of zero-thickness cohesive elements in the ITZ, and it is a new numerical approach. The results show that the model can simulate reasonably well the random damage pattern after RC beam load damage. The damage occurred in the middle of the beam span and tended to follow the ITZ. The model can predict the fatigue life of RC under various loads.

## 1. Introduction

With rapid economic development, the production of cars has increased, leading to the pollution of many waste tires, which are the primary source of waste rubber [1]. The combination of rubber, an excellent elastic material, and concrete, a brittle material, produces rubber concrete (RC), which has the advantages of low modulus of elasticity, high resistance to deformation, good crack resistance, good flexibility, and good wear resistance [2,3,4]. Liu et al. [2] found that RC improved concrete toughness and fatigue properties. Wang et al. [5] used the sounding technique to study the development of the fatigue damage process in RC at three stress levels—0.6, 0.7, and 0.8. It is a continuous process of the cumulative increase in damage, and it is divided into three processes: crack initiation, stable extension, and destabilization damage. With the rapid development of finite element theory and computer technology, concrete research is no longer limited to experimental studies. The method of finite element numerical simulation has become the primary research tool. Liu et al. [2] studied a mesoscale model of RC and analyzed its compressive properties. However, the model considered factors so simple that the results were unconvincing. Many scholars [6,7] have analyzed RC on microscopic, mesoscopic, and macroscopic scales, but no one has used a finite element model (FEM) to study RC fatigue. For this reason, this paper propose a finite element fatigue model of RC on the mesoscale.

Concrete mesoscale modeling studies have established aggregate, mortar, admixture, and interface transition zones (ITZ) over the last two decades [8]. Each component interacts with the others through mechanical relationships, thus influencing the strength of the overall structure. At the stage of concrete modeling, there are two ways of dealing with how to characterize concrete components. One technique is digital image technology recognition [9]. Zheng et al. [10] built a 2D concrete mesoscale model based on image recognition and investigated concrete’s compressive strength and dimensional effects. He provided a reliable method for predicting compressive strength. Second, by analyzing the concrete composition and using computer programming to create a random aggregate model (RAM) [11] that meets the requirements, Sharif et al. [12] simulated samples of biphasic cubic concrete containing spherical aggregates embedded in homogeneous mortar and successfully demonstrated the failure modes of the pieces. After the characterization of the mesoscale aggregate composition method is completed, there are two methods of computational modeling: one is an FEM based on a RAM [11], and the other is a mechanical model based on a discrete element model (DEM) [13]. P.S.M. et al. [14] successfully modeled finite element RAM of ultrahigh-strength concrete fracture under uniaxial compression. It was found that damage initiation may occur in any of the three phases on the mesoscale, a degree that is difficult to achieve by experimental means and DEM. In addition, Zhou et al. [15] built a three-point bending-notched concrete beam as a model structure to discuss the mechanism of crack sprouting. However, realistic concrete beams have no prefabricated cracks. This paper uses an FEM with RAM to model three-point bent concrete without prefabricated cracks for fatigue simulation.

FEM calculations are primarily based on elasticity mechanics [16], plasticity mechanics [17], damage mechanics [18], and fracture mechanics [19] theories. The elastic model treats concrete as an elastomer and studies the mechanical properties of concrete in its elastic range. The disadvantage of the elastic model is that it is challenging to study the properties of concrete after large deformation or cracking. The concrete-smeared cracking model [20] uses a linear elastic model, which makes it difficult to calculate non-linear forms of damage. Kim et al. [21] presented a plasticity model that considers the form of concrete damage and the area of damage. The mechanical model of concrete damage first evolved through the study of metal fatigue [22], which considered a concrete failure as a process of quantitative damage triggered by microcracks in mesoscale structures. Ray et al. [23] found that the influencing factor for concrete fatigue is size through fracture mechanics models analyzed on a macroscopic structure. Concrete damage form is not determined by one mechanical behavior but by various mechanical methods. This paper used the concrete damaged plasticity (CDP) model, which combined concrete elasticity, plasticity, and damage. The CDP model was first proposed by J. et al. [24], and then B. Xu et al. [25] presented a damage model for the cyclic loading of concrete structures. B. Xu et al. [25] found that this model can simulate the inelastic behavior of RC beam–column members very well. In this paper, plastic damage theory is used, and the model conforms to the requirements by improvement.

In a 2D mesoscale study, the RC components are mortar, aggregate, rubber, aggregate-mortar ITZ, and rubber-mortar ITZ. The ITZ is complicated, and its thickness is usually 10–50 μm [26], which exceeds the minimum size for numerical simulations. With the development of research in recent years, a method called a cohesive element (CE) [27] for dealing with damage to very small-thickness elements has been proposed. Wang et al. [28] investigated the effect of cohesion models on the tensile behavior of concrete. Zhao et al. [29] developed a crystal plasticity model combining an extended finite element approach with a CE model. They analyzed fatigue cracking and found that the simulations were consistent with previous experimental observations.

The purpose of this paper is to present a fatigue damage model applicable to RC. This model uses a new numerical simulation method. Different RC peak loads of static pressure and fatigue life were simulated using the CDP and CE models. Based on the model’s feasibility, the fatigue life of RC was predicted for different admixtures and loads, which can provide a basis for experimental reference in advance. Subsequent work can vary the load application methods, such as random and variable frequency loading, and can also consider a 3D mesoscale model study, which is of great significance.

## 2. Modeling Methods

The mesoscale model’s modeling approach begins by considering the geometry of the model generation, which includes aggregate content, size, and location. Subsequently, the constitutive model of concrete and the ITZ model is considered to make the model feasible.

### 2.1. Mesoscale Model Geometry Generation

In mesoscale studies, concrete is usually considered a three-phase material consisting of aggregate, mortar, and ITZ. The rubber particles in RC replace part of the fine aggregates. Concrete is regarded as a homogeneous material in conventional macroscopic concrete FEM. This visual modeling approach makes it difficult to investigate how the concrete’s inhomogeneity affects the macroscopic properties. This assumption of the homogeneity of concrete ignored several vital influences, such as aggregate size, particle size distribution, aggregate shape, and the effect of the ITZ. Zhong et al. [30] investigated the effect of aggregate shape (circular, elliptical, and polygonal) on the results of numerical analysis of the mesoscale model. They compared the stress–strain curves under different conditions with the experimental results. The results showed that the circular aggregate model is optimal for the numerical simulations. In this paper, circular aggregates are used so that meshing is easier and computer solutions are faster. In contrast, irregularly shaped aggregates are very complex to mesh and increase the computational burden.

In this paper, the coarse aggregates in the RC mesoscale model are aggregates of 5 mm or more in diameter and the fine aggregates are included in the mortar. The geometry of the mesoscale model needs to comply with three requirements: firstly, all the particles generated must be within the specified boundaries; secondly, none of the particles can overlap; and thirdly, there must be a gap between each particle, as the aggregates are wrapped in a layer of mortar and have no contact. Fuller’s particle size [31] distribution curves are used in this paper. The Fuller curve is widely regarded as the grading curve, which provides an optimum particle size distribution for the working condition of the concrete. The Fuller curve equation is as follows:

(1) $$P\;=100\sqrt{\frac{D_{0}}{D_{max}}}$$

where P represents the percentage of aggregate passing through sieve hole diameter *D*_0_, *D*_0_ represents the diameter of the sieve hole, and *D_max_* represents the diameter of the largest aggregate.

As this paper focuses on the 2D level, it is impossible to deal with the 2D problem directly with the help of the 3D Fuller set matching formula. It was used to obtain the best particle size distribution curve in 2D by applying the Walraven formula [32]. The formula is as follows:

(2) $$P_{c}\left(D<D_{0}\right)=P_{k}\left(1.065D_{0}^{0.5}D_{max}^{-0.5}-0.053D_{0}^{4}D_{max}^{4}-0.012D_{0}^{6}D_{max}^{-6}-0.0045D_{0}^{8}D_{max}^{-8}-0.0025D_{0}^{10}D_{max}^{-10}\right)$$

where *P*_c_ represents the percentage of the aggregate area, where size *D* is smaller than *D*_0_. P_k_ represents the percentage of the aggregate area of the total area. In this paper, *P*_k_ is taken to be 0.7. *D_max_* represents the diameter of the largest aggregate size, and the maximum diameter is taken as 20 mm.

The area of aggregate size distribution in the 550 mm × 150 mm area is listed by Formula (2) in Table 1.

**Figure 1:**
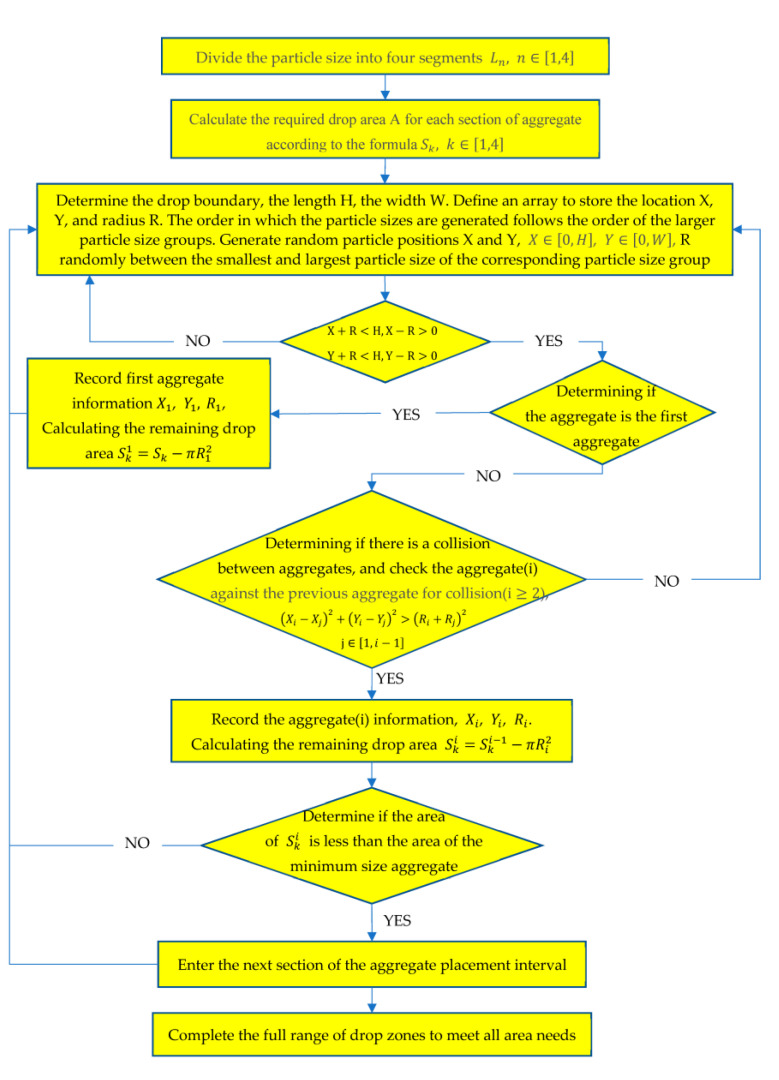
Flowchart of aggregate generation.

The random generation is implemented in Python according to the aggregate area in Table 1, and the generated flowchart is shown in Figure 1.

### 2.2. Constitutive Model of Concrete

The finite element software ABAQUS (2021 Version, Dassault systemes, France) and the programming language Python 3.8 are interconnected, and the code generated in Section 2.1 can be imported directly into ABAQUS. Aggregates are developed according to the program, and different property values are assigned to the different components to achieve the actual state of the mesoscale RC aggregates.

**Figure 2:**
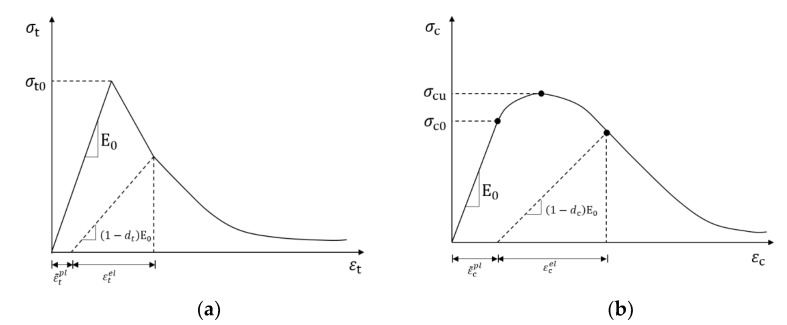
The uniaxial stress–strain response: (**a**) tensile; (**b**) compressive.

In this paper, coarse aggregate and rubber are considered homogeneous elastomers, and mortar is modeled numerically as a homogeneous continuum with elasticity. The mortar can be regarded as a lower-strength type of concrete, and its constitutive law uses the concrete–damage–plasticity (CDP) model. The CDP is a continuous, plasticity-based damage model that defines the concrete state by defining two mechanical behaviors: tensile cracking and compression damage. This model assumes that the concrete’s uniaxial tensile and compressive response is characterized by plastic damage. The evolution of the yield surface is controlled by two hardening variables, the tensile equivalent plastic strain ${\tilde{\varepsilon }}_{t}^{pl}$ and the compressive equivalent plastic strain ${\tilde{\varepsilon }}_{c}^{pl}$, which are related to the damage mechanisms under tensile and compressive loading. The uniaxial tensile and compressive stress–strain response is shown in Figure 2.

Where ${\tilde{\varepsilon }}_{t}^{pl}$ and ${\tilde{\varepsilon }}_{c}^{pl}$ represent the equivalent plastic strains in tension and compression, $\varepsilon_{t}^{el}$ and $\varepsilon_{c}^{el}$ represent the elastic strains corresponding to tension and compression, $d_{t}$ and $d_{t}$ represent the two damage variables for elastic stiffness degradation, with the damage variables taking values from 0 to 1, and d=0 means the material is undamaged, d=1 means the material is completely damaged, and $E_{0}$ represents the initial Young’s modulus of the material.

In the case of uniaxial tension, the concrete stress–strain response obeys linear elastic variation up to the time of failure stress $\sigma_{t0}$, which is used to distinguish between the elastic and plastic phases of concrete. After $\sigma_{t0}$, the concrete enters the damage phase, and microcracking occurs in the macrostructure. In the case of uniaxial compression, the concrete stress–strain response obeys a linear elastic change to the compressive elastic ultimate stress, and $\sigma_{c0}$, and $\sigma_{c0}$ used to distinguish the elastic phase from the plastic phase under uniaxial compression. Unlike uniaxial tension, there is a hardening phase to the ultimate compressive stress $\sigma_{cu}$ after $\sigma_{cu}$ where the concrete is softened and microcracked.

When a concrete specimen is unloaded from any point in the strain-softening branch of the stress–strain curve, the elastic stiffness of the material appears to be damaged. The stress–strain relationships for uniaxial tensile and compressive loading are (Equation (3)):

(3) $$\sigma_{t}=\left(1-d_{t}\right)\;E_{0}\;\left(\varepsilon_{t}-{\tilde{\;\varepsilon }}_{t}^{pl}\right)$$

(4) $$\sigma_{c}=\left(1-d_{c}\right)\;E_{0}\;\left(\varepsilon_{c}-{\tilde{\varepsilon }}_{c}^{pl}\right)$$

**Figure 3:**
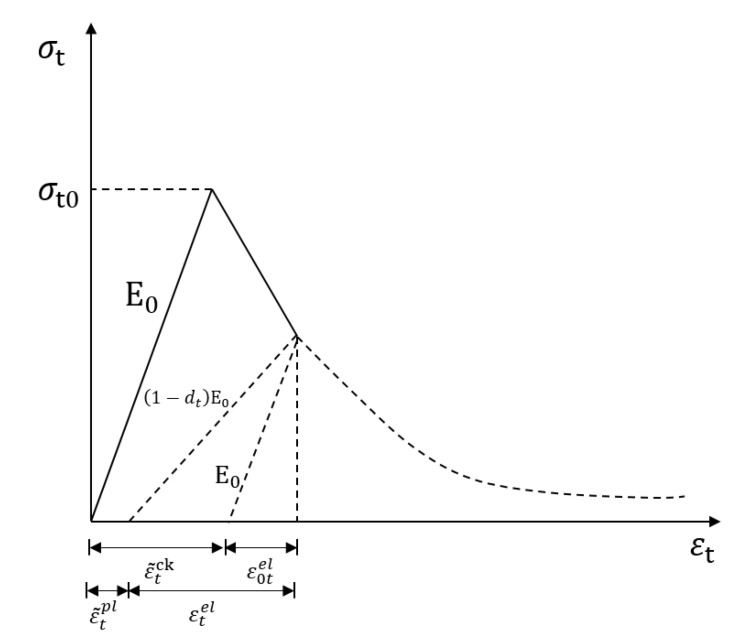
Redefinition of tensile stiffness after damage.

This paper deals with the numerical simulation of the three-point bending of concrete, where the general form of damage is tensile damage. After being subjected to cyclic loading, the tensile stiffness after damage needs to be redefined, as shown in Figure 3.

Where $\varepsilon_{0t}^{el}$ represents elastic strain and ${\tilde{\varepsilon }}_{t}^{ck}$ represents cracking strain.

In the event of damage to the concrete, the cracking strain ${\tilde{\varepsilon }}_{t}^{ck}$ is defined by the following equation:

(5) $${\tilde{\varepsilon }}_{t}^{ck}=\varepsilon_{t}-\varepsilon_{0t}^{el}$$

(6) $$\varepsilon_{0t}^{el}=\frac{\sigma_{t}}{E_{0}}$$

ABAQUS automatically converts cracking strain to plastic strain for use:

(7) $${\tilde{\varepsilon }}_{t}^{pl}={\tilde{\varepsilon }}_{t}^{ck}-\frac{d_{t}}{\left(1-d_{t}\right)}\;\frac{\sigma_{t}}{E_{0}}$$

According to the Structural Design Code for Concrete [33], considering the tensile and compressive damage variables of the material, the specific concrete intrinsic model is determined by Young’s modulus E and Poisson’s ratio λ in the elastic phase and by the non-linear stress–strain equation in the inelastic phase. When the concrete structure is under pressure:

(8) $$\sigma_{c}=\left(1-d_{c}\right)\;E\varepsilon_{c}$$

(9) $$d_{c}=\left\{\begin{array}{c} 1\;-\;\frac{\rho_{c}\;n}{n\;-\;1\;+\;x^{n}}\;x\leq 1\\ 1-\frac{\rho_{c}\;}{\alpha_{c}{\left(x\;-\;1\right)}^{2}\;+\;x}x>1 \end{array}\right.$$

(10) $$\rho_{c}=\frac{f_{c,r}}{E_{c}\varepsilon_{c,r}}$$

(11) $$x=\frac{\varepsilon }{\varepsilon_{c,r}}$$

(12) $$n=\frac{E_{c}\varepsilon_{c,r}}{E_{c}\varepsilon_{c,r}-f_{c,r}}$$

where $\alpha_{c}$ is the parameter value of the falling section of the uniaxial compressive stress−strain curve for concrete, $f_{c,r}$ is the representative value of the uniaxial compressive strength of concrete, $\varepsilon_{c,r}$ is the peak compressive strain corresponding to $f_{c,r}$, and $d_{c}$ is the evolutionary parameter for uniaxial compressive damage to concrete.

When the concrete structure is in tension:

(13) $$\sigma_{t}=\left(1-d_{t}\right)E\varepsilon_{t}$$

(14) $$d_{t}=\left\{\begin{array}{c} 1-\rho_{t}\;\left[1.2\;-\;0.2x^{5}\right]x\leq 1\\ 1-\frac{\rho_{t}}{\alpha_{t}{\left(x\;-\;1\right)}^{1.7}\;+\;x}\;x>1 \end{array}\right.$$

(15) $$x=\frac{\varepsilon }{\varepsilon_{t,r}}$$

(16) $$\rho_{t}=\frac{f_{t,r}}{E_{c}\varepsilon_{t,r}}$$

where $\alpha_{t}$ is the parameter value of the falling section of the uniaxial tension stress−strain curve for concrete, $f_{t,r}$ is the representative value of the uniaxial tension strength of concrete, $\varepsilon_{t,r}$ is the peak tension strain corresponding to $f_{t,r}$, and $d_{t}$ is the evolutionary parameter for uniaxial tension damage to concrete.

In accordance with the Structural Design Code for Concrete [33], specific values are shown in Table 2.

In addition to this, the plasticity parameters for CDP are self-contained in ABAQUS, as shown in Table 3.

The values in Table 3 have been verified by many academics to be generally consistent. This is a fixed value [10].

### 2.3. CE Model of the ITZ

After the aggregate model has been built, there are two approaches to the ITZ. One is establishing a solid FEM of the ITZ [34]. The advantage of this is that it can reflect the thickness relationship of the interface composed of concrete. However, the ITZ’s actual thickness is 10–50 μm, which is difficult to achieve with FEM. Even if the thickness is expanded by a factor of 10 to a range that FEM can calculate, this will result in a dense and small mesh division and a significant increase in computational effort. Secondly, the ITZ is considered a zero-thickness element (ZTE) [35], which retains the relevant mechanical properties of the actual ITZ to achieve the accuracy of the simulation, and all ITZs in concrete can be represented by ZTE. In summary, we selected the ZTE.

**Figure 4:**
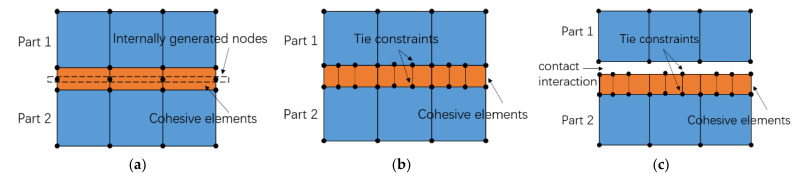
Three types of CE: (**a**) shared nodes; (**b**) tie constraint; (**c**) contact interaction.

The ZTE has three ways of simulating the behavior of the ITZ. Firstly, a layer of the ZTE can be inserted using a shared node, which can be used if the CE is on the same mesh as the surrounding element. Secondly, if the elements of the CE are divided differently from the surrounding mesh or if the CE uses a finer discretization than the adjacent parts, the tie constraint can be used to connect the CE to other parts. Thirdly, in some special cases where the requirements are met, a connected interaction can be added directly to the CE in contact without adding additional elements. Figure 4 shows the three methods of CE processing.

**Figure 5:**
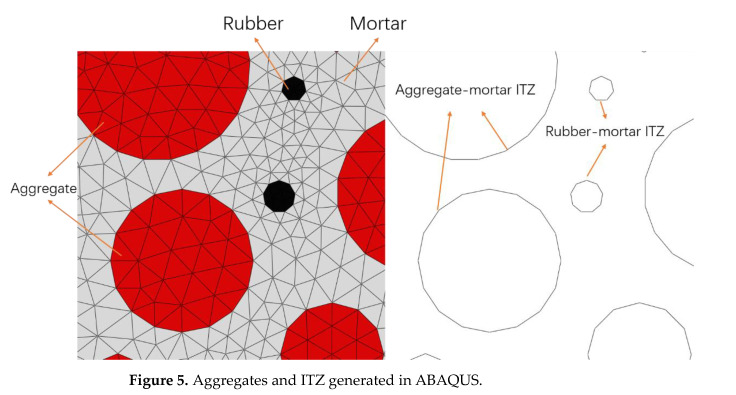
Aggregates and ITZ generated in ABAQUS.

Based on the random aggregates generated by the simulations in this paper, a cohesive zone will be added to the contact surface of the aggregate and mortar. The first ZTE (Figure 4a) is chosen to insert a layer of CE using a shared node. The size and location of each aggregate are uncertain, so choosing inserted CE is difficult. A Python program finds the node number of the aggregate place and copies the new node at the node number to create a zero-thickness CE. This fits perfectly with the shared node insertion approach. The ITZ generates CE, as shown in Figure 5.

The CE damage is divided into four parts: the linear elastic phase, the damage initiation phase, the damage evolution phase, and complete damage.

The online resilience phase of the damage response of the CE is as follows:

(17) $$t\;=\left\{\begin{array}{c} t_{n}\\ t_{s}\\ t_{t} \end{array}\right\}=\left[\begin{array}{c} E_{nn}\;E_{ns}\;E_{nt}\\ E_{ns}\;E_{ss}\;E_{st}\\ E_{nt}\;E_{st}\;E_{tt} \end{array}\right]\;\left\{\begin{array}{c} \varepsilon_{n}\\ \varepsilon_{s}\\ \varepsilon_{t} \end{array}\right\}=E\varepsilon$$

where $t_{n}$ is the nominal stress in the normal direction, $t_{s}$ is the nominal stress in shear in the first direction, $t_{t}$ is the nominal stress in shear in the second direction, $E_{ij}$ is Young’s modulus in each direction, and $\varepsilon_{i}$ is the strain in the corresponding direction.

The quadratic stress criterion formula is used for damage initiation. Damage initiation occurs when the contact-stress ratio involved reaches 1:

(18) $${\left\{\frac{t_{n}}{t_{n}^{0}}\right\}}^{2}+{\left\{\frac{t_{s}}{t_{s}^{0}}\right\}}^{2}+{\left\{\frac{t_{t}}{t_{t}^{0}}\right\}}^{2}=1$$

**Figure 6:**
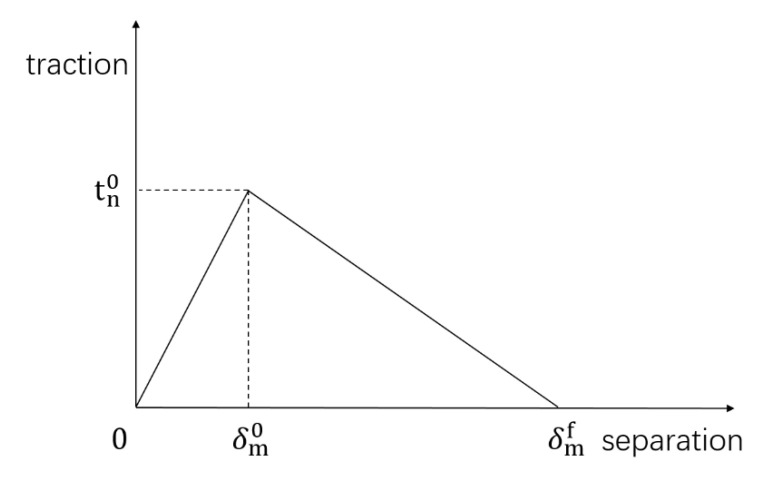
Traction separation relationship.

Damage evolution by way of traction separation is shown in Figure 6.

Where $\delta_{m}^{0}$ is the separation displacement value at the onset of damage and $\delta_{m}^{f}$ is the separation displacement at the maximum damage.

The material enters the damage phase judged by the damage value D. When D is 1, the material is completely damaged by the following equation:

(19) $$D\;=\frac{\delta_{m}^{f}\;\left(\delta_{m}^{max}-\delta_{m}^{0}\right)}{\delta_{m}^{max}\;\left(\delta_{m}^{f}-\delta_{m}^{0}\right)}$$

where D is the damage value and $\delta_{m}^{max}$ is the additional amount of maximum separation displacement during loading.

In this paper, the fracture energy is calculated using the Benzeggagh–Kenane (BK) criterion:

(20) $$G^{C}=G_{n}^{C}+\left(G_{s}^{C}-G_{n}^{C}\right)\;{\left\{\frac{G_{s}}{G_{T}}\right\}}^{\eta }$$

where $G^{C}$ is the hybrid fracture energy, $G_{n}^{C}$ is the type I fracture energy of the cohesive element, $G_{s}^{C}$ is the type II fracture energy of the cohesive element, $G_{s}$ is the shear deformation energy, and $G_{T}$ is the tensile deformation energy.

The performance of ITZ is difficult to test on the experimental scale, so the determination of simulation parameters for ITZ is difficult to determine. Usually, the performance of ITZ is approximated by the weak mortar composition, and researchers use the percentage of mortar to study and judge the performance of ITZ. Xiao et al. [36] considered the strength of ITZ to be 80% of the mortar. Kim et al. [37] considered the fracture energy of ITZ to be equivalent to 50% of the mortar. Li et al. [38] considered it to be 80%. It was obvious that different researchers have different opinions on determining the mechanical properties of ITZ. The ultimate purpose is to achieve unity between numerical simulations and experiments, so the performance parameters of the ITZ on numerical simulations are determined by trial and error to determine the optimum values of these relevant parameters. The parameters used in this paper are shown in Table 4.

## 3. Verification of the Model

### 3.1. Experiment

The data for this summary test were obtained from Liu et al. [39]. He investigated the effect of rubber substitution rate and rubber particle size on the fatigue life of rubber concrete. The object of study was an RC beam with dimensions of 150 mm × 150 mm × 550 mm. A static load test was conducted under a three-point bending load, and a fatigue test was conducted under a cyclic load. The fatigue life and related fatigue life curves were obtained for different rubber substitution rates, particle sizes, and stress levels.

#### 3.1.1. Experimental Materials

Material parameters are shown in Table 5 and Table 6.

#### 3.1.2. Experimental Test Methods

RC specimens with different rubber replacement rates are first tested by static loading to obtain the corresponding peak loads. The fatigue tests are carried out using models of the same material proportions. The maximum and minimum loads are applied to the RC beams using a load-controlled mode, which is an equal amplitude and uniform load mode.

### 3.2. Building Mesoscale Models

**Figure 7:**
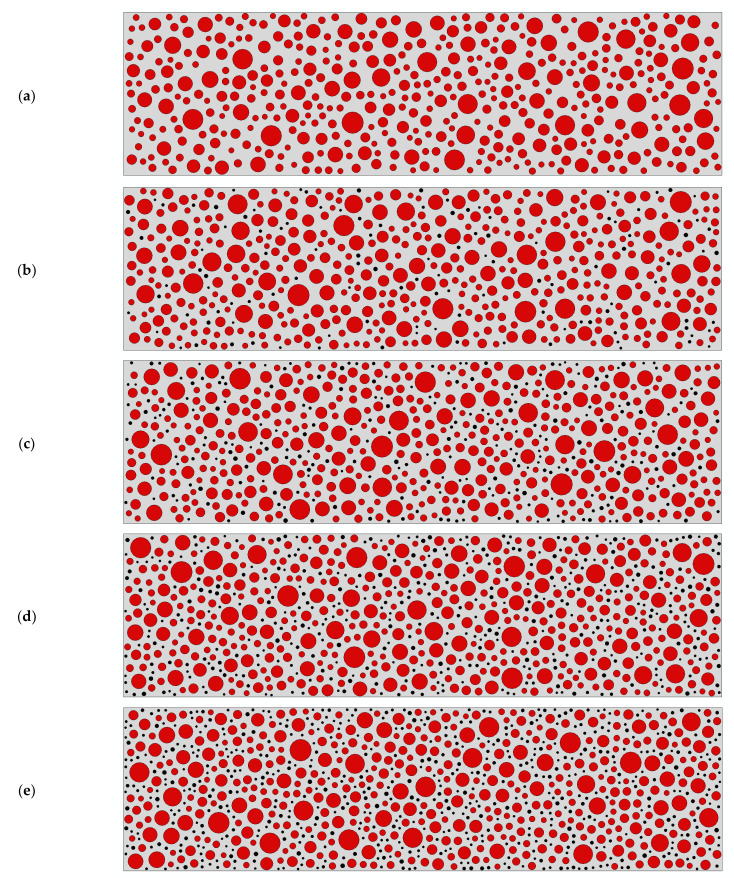
RC beams with different dosing: (**a**) 0; (**b**) 2.5%; (**c**) 5%; (**d**) 7.5%; (**e**) 10%.

A 150 mm × 550 mm rubber concrete beam element is built according to Table 1, with different dosing of rubber concrete beams as shown in Figure 7, where gray means mortar, red means aggregate, and black means rubber.

**Figure 8:**
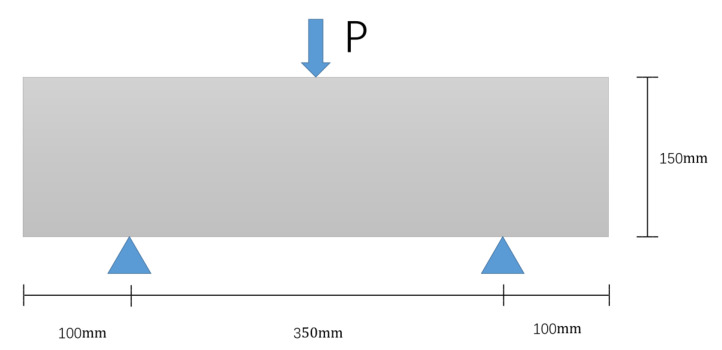
Load loading schematic.

The load loading point is in the middle of the upper part, with the bottom left constraint 100 mm from the left boundary and the right constraint 100 mm from the right, as in Figure 8.

The model is solved using the ABAQUS/Standard. First, apply a displacement constraint of 2 mm at the upper load loading point, stop the calculation when the model does not converge, and obtain the peak load $F_{max}$ for the model. Subsequently, the fatigue life of the model is calculated at different stress levels, still using the same model with cyclic concentrated force constraints applied at the upper load loading points. Load application from minimum load $P_{min}$ to maximum load $P_{max}$, where $P_{min}/P_{max}\;$= 0.1, fatigue load stress levels S = $P_{max}/F_{max}$. In this paper, S takes the values 0.9, 0.85, 0.8, and 0.75. When S is too high, the fatigue damage results are over in one go. When S is too small, the calculation is too large in the numerical simulation phase. The Fourier series method controls the equivalent mean amplitude load when fatigue loads are applied:

(21) $$F\left(t\right)=\left\{\begin{array}{c} A_{0}+\sum_{n}^{N}\left[A_{n}\cos n\omega \left(t-t_{0}\right)+B_{n}\sin n\omega \left(t-t_{0}\right)\right]t\geq t_{0}\\ \;A_{0}0\leq t\leq t_{0} \end{array}\right.$$

where the period is T, circle frequency ω = 2π/T, the loading initial time is $A_{0}$, and the number of steps parameters $A_{1},B_{1},A_{2},B_{2},\cdot \cdot \cdot ,A_{0},A_{0}$.

The parameters used for RC in this paper are shown in Table 7.

### 3.3. Experimental Versus Simulation

By comparing the results of this study with the three-point bending static load peak load results and fatigue load results from the literature [39], the feasibility of the model is verified.

#### 3.3.1. Peak Load

The peak load tests and simulation results for this model under three-point bending loads at different stress levels are summarized in Table 8. It can be seen that the magnitude of the peak load decreases as the rubber content increases, which is in line with the researchers’ judgment on the performance of RC. Comparing the test and simulation for peak loads at the same stress levels proved that the simulation and test results agree well. The maximum absolute error is 3.6%. The new numerical model proved reliable for peak loads under three-point bending loads.

#### 3.3.2. Fatigue Life

**Figure 9:**
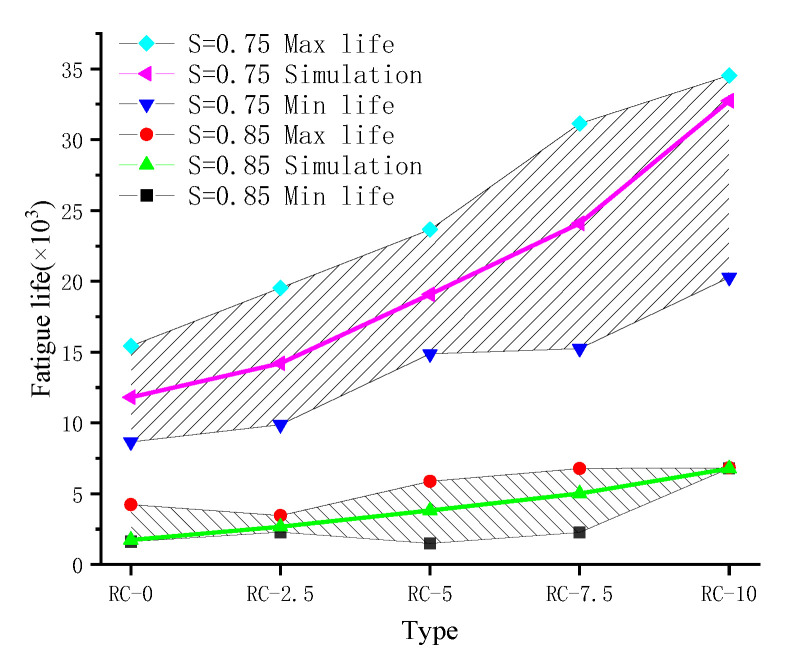
Experimental and simulated fatigue life.

The results of tests and simulations with different dosing levels of rubber concrete at stress levels S = 0.85 and S = 0.75 are summarized in Table 9. The increase in rubber admixture can improve the fatigue resistance of RC and extend the fatigue life. Due to the large dispersion of the fatigue life results, only the minimum and maximum lives are taken as a reference in the test results. Moreover, the overall life trend improves with increasing rubber doping. As shown in Figure 9, the results obtained from the numerical model of rubber concrete in this paper are all between the maximum and minimum values of the test results and meet the feasibility requirements of the model. This proves the reliability of the new numerical model in fatigue life calculation. There are some differences between the expected life and the experiment, but this is acceptable. Because the experiment phase is a one-off for each test beam, the RC is already destroyed after the experiments with peak load. Although each beam is made to the same size and aggregate content, the mechanical properties are not the same. The different mechanical behaviors of the concrete beam can be observed in [37].

## 4. Analysis of Variables

Finite element static pressure simulations of three-point bending were carried out for RC rubber admixtures of 0, 2.5%, 5%, 7.5%, and 10% to obtain the corresponding peak load and displacement relationships. Based on the stress levels S = 0.85 and S = 0.75 above, add stress levels S = 0.9 and S = 0.8 to the loading method for the RC fatigue simulation to analyze the effect of different rubber doping and stress levels on damage form and fatigue life.

### 4.1. Force–Deflection Curves

**Figure 10:**
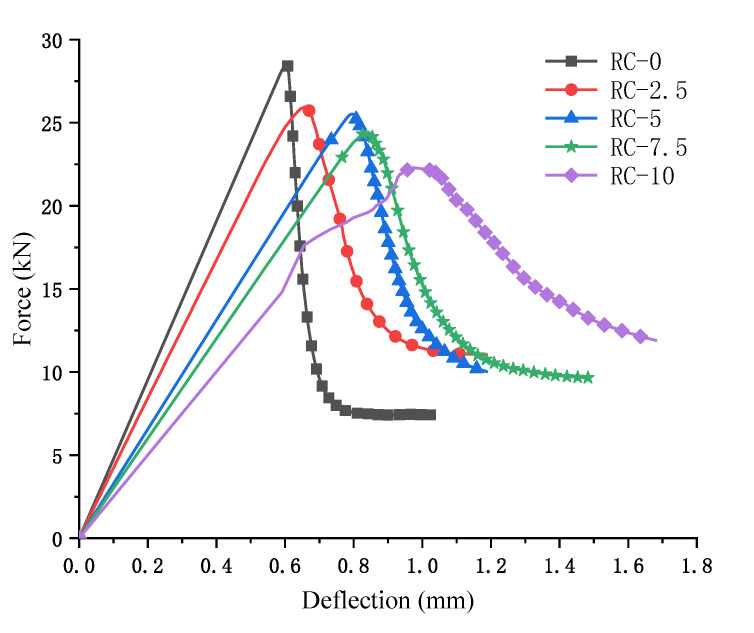
Load–deflection curve.

The force–deflection curves for RC at different admixtures were obtained from numerical simulations, as shown in Figure 10. The concrete deflection increases as the rubber admixture increases and the peak load tends to decrease significantly. The rising and falling phases of the curve for ordinary concrete are steeper than the gentle curve for RC. The trend becomes more subdued as the amount of rubber added increases. The comparison of the trends of the two curves RC-0 and RC-10 in Figure 10 is exceptionally different. It confirmed the effect of rubber particles on concrete in the mesoscale study. Rubber was able to reduce the extension of concrete damage and increase the toughness of concrete, reducing the brittleness of concrete. This reflects the actual validity of the new numerical model.

### 4.2. Types of Damage

**Figure 11:**
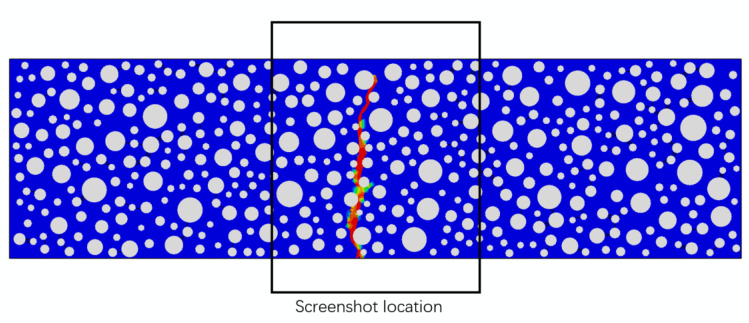
Damage to ordinary concrete and location of damage sampling.

In this paper, the damage to the RC is shown through stiffness in the form of two factors: one is static compression, and the other is fatigue. The visual form of the damage is represented by the SDEG cloud map output by ABAQUS (SDEG = 0 means no damage to the structure, and SDEG = 1 means complete damage to the structure). Figure 11 shows the damage to an ordinary concrete beam of 150 mm × 550 mm without rubber admixture after a three-point bending static load. As the beam damage occurs in the middle of the span, for ease of observation, the structure is taken in the middle of the beam, as shown in the black box in Figure 11, with a size of 150 mm × 150 mm. The following are screenshots of the damage obtained by this method.

**Figure 12:**
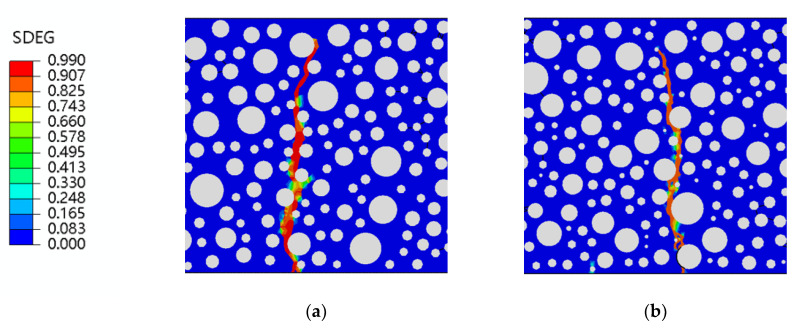

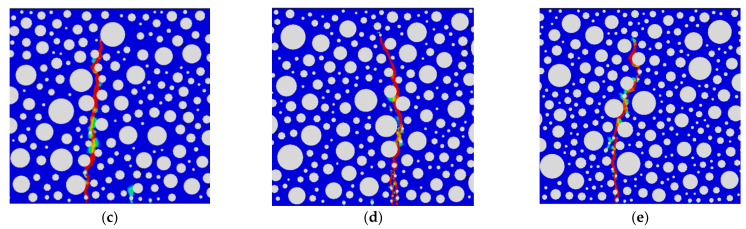
RC SDEG with different mixes for static pressure loading: (**a**) 0; (**b**) 2.5%; (**c**) 5%; (**d**) 7.5%; (**e**) 10%.

The SDEG damage clouds for 0, 2.5%, 5%, 7.5%, and 10% rubber doping after damage are shown in Figure 12. A form of static pressure damage to rubber concrete was observed in the mesoscale study. The damage was mainly at the mid-span of the beam, with an irregular damage zone extending from the bottom to the top. The damage course follows the edges of the aggregate and rubber and is consistent with existing fracture and damage mechanics theories. The point of damage to the zero rubber-doped concrete is only at the opening of the damage zone, with no damage to the surrounding concrete aggregate, as shown in Figure 12a. Damage points occur not only at the opening of the damage zone but also minor damage to the rubber around the opening, as shown in Figure 12b–e. On the mesoscale, it is observed that the rubber particles take up a small part of the load-bearing capacity under load. Furthermore, with the increase of rubber admixture, the damage point at the bottom of the concrete increases, and the damage zone is influenced by the surrounding rubber particles in the middle of the extension. The 10% and 7.5% rubber-doped concrete leads particularly well, with multiple damage points at the bottom and tiny branches of the damage zone midway through, as shown in Figure 12d,e. Various forms of damage indicate that adding rubber particles to concrete helps to retard concrete damage, which also provides the basis for research into the fatigue resistance of RC.

**Figure 13:**
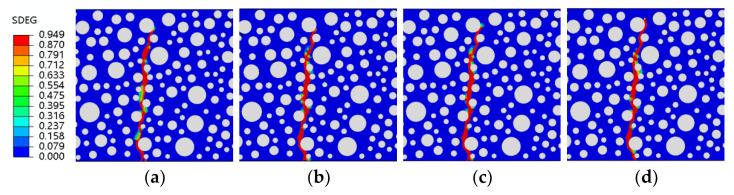
SDEG of ordinary concrete under different stress levels: (**a**) S = 0.9; (**b**) S = 0.85; (**c**) S = 0.8; (**d**) S = 0.75.

The fatigue simulation of the same RC beam at different stress levels is a unique advantage of the fine-view simulation. The stress levels are guaranteed to be the same peak load each time, something that cannot be achieved experimentally. This paper uses fatigue simulations for four stress levels of 0.9, 0.85, 0.8, and 0.75, with each stress level corresponding to five rubber doping levels. Shown in Figure 13 are four forms of stress level fatigue damage for ordinary concrete. It can be observed that fatigue damage to ordinary concrete at different stress levels takes the same form, with the damage zone starting at the same point of failure at the bottom of the concrete. In ordinary concrete, from the start of the damage to the end, only the weakest point within the concrete bears the load, regardless of the force acting. Its fatigue damage is also essentially the same as static pressure damage (Figure 12a and Figure 13), proving that ordinary concrete is relatively homogeneous regarding internal forces when damaged, with the same place bearing the load.

**Figure 14:**
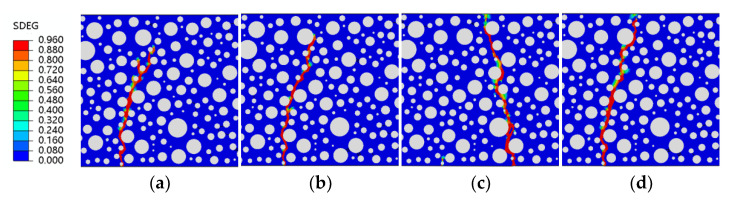
SDEG of RC-2.5 under different stress levels: (**a**) S = 0.9; (**b**) S = 0.85; (**c**) S = 0.8; (**d**) S = 0.75.

**Figure 15:**
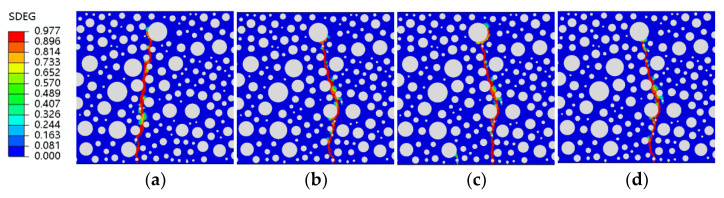
SDEG of RC-5 under different stress levels: (**a**) S = 0.9; (**b**) S = 0.85; (**c**) S = 0.8; (**d**) S = 0.75.

**Figure 16:**
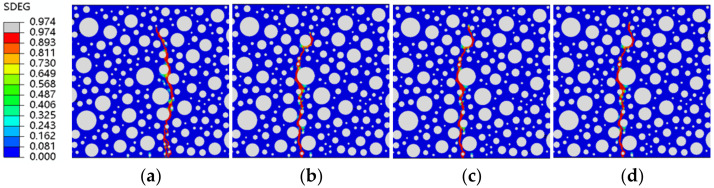
SDEG of RC-7.5 under different stress levels: (**a**) S = 0.9; (**b**) S = 0.85; (**c**) S = 0.8; (**d**) S = 0.75.

**Figure 17:**
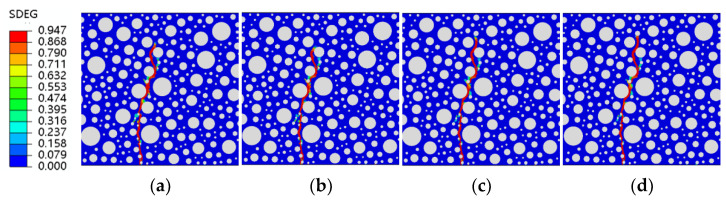
SDEG of RC-10 under different stress levels: (**a**) S = 0.9; (**b**) S = 0.85; (**c**) S = 0.8; (**d**) S = 0.75.

Figure 14, Figure 15, Figure 16 and Figure 17 show fatigue damage at four stress levels for four doped RC. Unlike ordinary concrete, the fatigue loads do not take the same form of damage at different stress levels when rubber is added. As shown in Figure 14, the damage zone for 2.5% admixture stress levels of 0.85, 0.8, and 0.75 differ, and the damage point is also different at the bottom of the concrete. As shown in Figure 15, the location of the damage zone is different for 5% doping stress levels of 0.9, 0.85, and 0.8, but the location of the bottom damage point is the same for stress levels of 0.85, 0.8, and 0.75. As shown in Figure 16, the 7.5% doping stress level only differs in the damage zone and damage at a stress level of 0.9; the damage zone and damage point are essentially the same at other stress levels. As shown in Figure 17, the orientation of the damage zone and the location of the initial damage point at the bottom stabilize and remain the same when the doping level reaches 10%. The rubber dosing ranges from 0 to 10%, with the damage zone orientation and initial damage point location stabilizing from the beginning, through the disorder of the intermediate dosing, and to final stability. The fatigue damage of RC is different from hydrostatic damage, which is also different from ordinary concrete. The addition of the rubber creates a fragile ITZ between the rubber and the mortar, even weaker than the ITZ between the aggregate and the mortar. The model successfully simulated the effect of rubber doping.

### 4.3. Fatigue Life

Table 10 shows the peak loads and the life of the fatigue loads at the corresponding four stress levels for the five doped rubber concretes. At the same stress level, the fatigue life increases as the rubber content increases, indicating that rubber concrete carries higher cyclic loads than ordinary concrete for a given cyclic load. In the case of rubber concrete, this result is because the rubber particles act as an energy absorber and load cushion in the concrete. Rubber particles have better elastic properties on the mesoscale level than concrete particles. In the case of concrete suffering from tension and compression, part of the energy is converted into the elastic energy of the rubber particles. The fatigue life of 10% rubber is 7.3, 3.89, 4.45, and 2.77 times greater than that of ordinary concrete at four stress levels.

**Figure 18:**
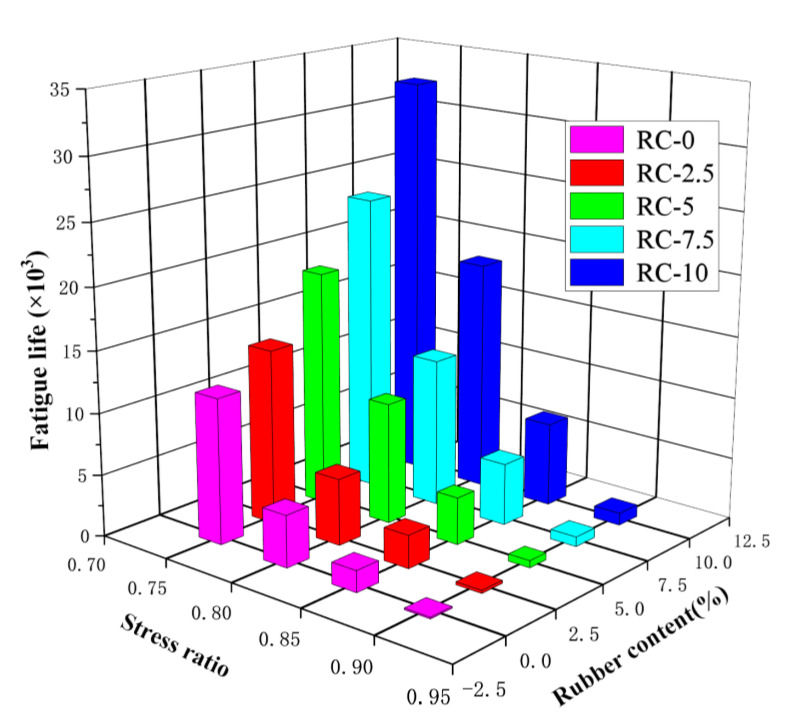
Fatigue life of different rubber doping at different stress levels.

**Figure 19:**
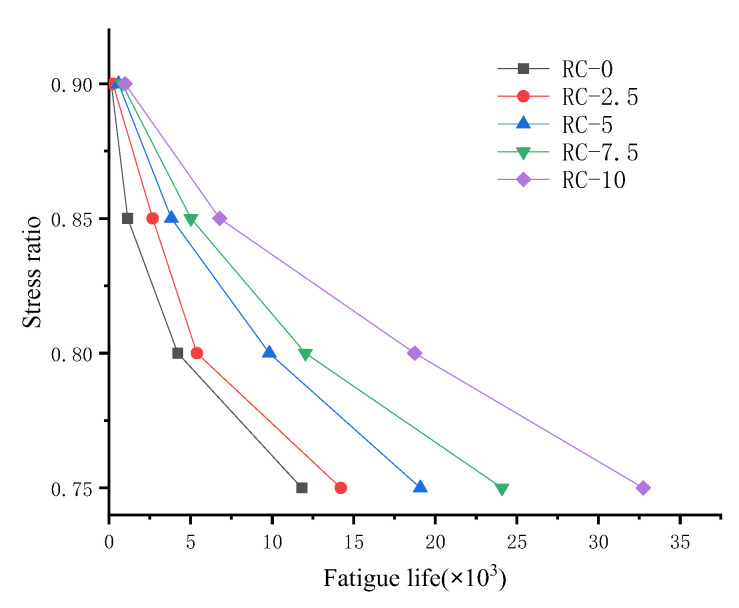
Relationship between stress level and fatigue life of different rubber doping.

The relationship between rubber doping, stress level, and fatigue life is shown in Figure 18. It is obvious that, within a specific range, an increase in rubber content and a decrease in stress level increase the fatigue life of RC. The increase in fatigue life is a non-linear relationship, as shown in Figure 19.

## 5. Discussion

This model simulation study generates the mesoscale structure of RC through random aggregates, applies the improved properties of CDP to mortar, and combines modeling of the aggregate-mortar ITZ and rubber-mortar ITZ to achieve the mesoscale structure of actual RC. It is a new numerical approach. The model was subjected to a series of three-point bending fatigue loads to analyze the causes of damage forms and fatigue life from a mesoscale.

### 5.1. Causes of Damage Types

The structural form of the model after damage by static pressure and fatigue loading is consistent with reality, and the appearance of concrete damage on the mesoscale can be accurately observed from the mesoscale structure. Subsequent damage develops along the weakness of the ITZ around the aggregate and rubber particles. There are two main reasons for producing an irregular damage band consistent with reality. First, the model adds the damage theory in the CE model, setting a zero-thickness damage zone in the aggregate-mortar and rubber-mortar layers. The material’s mechanical properties in the CE are less than those of the mortar. When subjected to forces, the ITZ is more easily damaged than the mortar aggregate. Secondly, the mesoscale model is randomly generated for aggregate size and location, which aligns with the actual aggregate distribution of concrete materials and better reflects the model’s realism. The rubber particles are smaller than the coarse aggregate, and it is easier for damage to occur around the rubber than around the coarse aggregate.

### 5.2. Factors Influencing Fatigue Life

The addition of rubber benefits the fatigue properties of rubber concrete. When the model is damaged, there is some damage around the rubber particles not in the damage zone. These share some of the fatigue load, confirming the effect of the rubber particles on the mesoscale level. The mesoscale model can be applied to complex fatigue loads. The model shows fatigue life agreement at stress levels of 0.75 to 0.9 and can simulate the effects of fatigue life due to different doping levels of rubber. Rubber particles have better elastic properties on the mesoscale level than concrete particles. When the concrete is loaded, part of the energy is converted into the elastic energy of the rubber particles. RC life increases with increasing rubber and decreases with increasing stress ratio. As a rule of thumb, the magnitude of the stress ratio is related to the logarithm of the fatigue life [40]. After trying various fitting formulae, the following relationship is assumed:

(22) $$S\;=\;A\;+\;\mathrm{Bln}\left(N\right)+{\;\mathrm{Cln}}^{2}\left(N\right)\;\left(N>e^{-\frac{B}{2C}}\right)$$

where S is the concrete stress ratio, A, B, and C are constants whose magnitude is related to the concrete admixture, and N is the concrete fatigue life.

**Figure 20:**
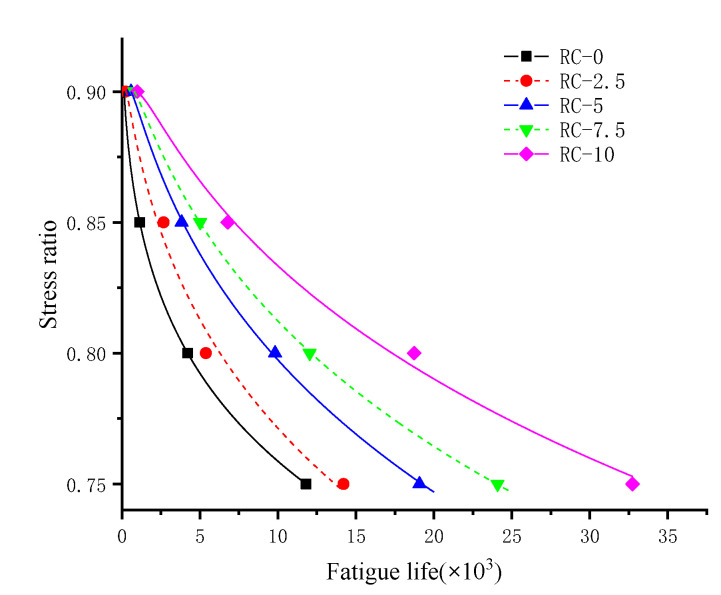
Fitting relationship between different rubber doping stress levels and fatigue life.

According to Formula (22). for curve fitting, as shown in Figure 20, the specific formula and correlation coefficient R results are shown in Table 11, and the fitted results meet the requirements. It is found that the stress level is related to the quadratic function of the logarithm of fatigue life. In addition, the results of this study allow for reasonable extrapolation of the three-point bending fatigue life of rubber concrete at dosing levels between 0.75 and 0.9. This provides a corresponding reference for the test, a model for calculating fatigue life correctly on the mesoscale.

### 5.3. Potential Applications and Developments

The mesoscale model proposed in this study can accurately represent the fatigue life of rubber concrete under three-point bending fatigue loading. In addition, analysis of the static pressure load’s damage form and the RC’s ultimate load reveals that the model is also accurately represented. The study of RC is equally informative for ordinary concrete and other polymer admixture concrete. The difference lies in the polymer’s shape, size, location, and properties. Shape, size, and position are solved by Python code, and performance could be solved by setting properties on the polymer. However, it is often difficult to achieve the desired effect, and an interface layer is needed to change the mechanical relationship between the different substances. This study could also be applied to reinforced RC to simulate the location of damage and structural life of specific damaged structures in macrostructures to provide an initial structural performance judgment for actual structures.

The limitations of this research method lie in the 2D structure. When considering the mesoscale design in the 3D structure, the lack of computer performance is challenging to resolve, and the vast number of calculations leads to increased calculation time. Future work could be improved to develop a 3D mesoscale concrete model to calculate fatigue life, achieving the desired accuracy and computational efficiency requirements.

## 6. Conclusions

This paper proposed an RAM on the scope of a mesoscale study. The model used plastic damage theory and the insertion of cohesive elements in the ITZ, and is a new numerical model. This paper verifies the model’s correctness in peak load and fatigue life. Peak loads were verified for five doping levels of 0, 2.5%, 5%, 7.5%, and 10%, and fatigue life was verified for stress levels of 0.75 and 0.85. After this, the results for stress levels of 0.8 and 0.9 were simulated and analyzed. The peak static pressure load in three-point bending was successfully modeled on a mesoscale as decreasing with increasing rubber doping, and the resulting deflection increased with increasing rubber doping. Static pressure and fatigue forms of damage could be observed in the mesoscale, where the point of damage produced by RC damage is not unique and increases with the amount of rubber admixture. The damage element produced by RC damage shows an order–disorder–order process as the rubber dosing increases. It was observed from the model that when the damage occurred to the RC, the internal rubber took the load. In addition, the model could simulate the three-point bending fatigue life at different stress levels for various rubber doping on a mesoscale. A quadratic function relating stress levels with different rubber doping to fatigue life was fitted, which can predict fatigue life for stress levels between 0.75 and 0.9, providing some reference value for the test. The mesoscale model in this paper satisfied the fatigue life simulation requirements perfectly. The method, with model improvements, could be applied to all RC fatigue structures in the future.

## Figures and Tables

**Table 1:** Size in 550 mm×150 mm area occupied by different particle sizes.

Rubber Replacement Rate (%)	5–10 mm (mm^2^)	10–15 mm (mm^2^)	15–20 mm (mm^2^)	Rubber (mm^2^)
0	5612	8851	12,547	0
2.5	5612	8851	12,547	768
5	5612	8851	12,547	1537
7.5	5612	8851	12,547	2305
10	5612	8851	12,547	3074

**Table 2:** Parameters of CDP.

$f_{c,r}\;(\mathrm{MPa})$	$\varepsilon_{c,r}\;\left(\times 10^{-6}\right)$	$\alpha_{c}$	$f_{t,r}(\mathrm{MPa})$	$\varepsilon_{t,r}\;\left(\times 10^{-6}\right)$	$\alpha_{t}$
40.26	1790	1.947	3.01	84	2.831

**Table 3:** Parameters of ABAQUS itself.

Dilation Angle	Eccentricity	$f_{b0}/f_{c0}$	K
30	0.1	1.16	0.666

**Table 4:** Parameters of ITZ.

	Normal Strength (MPa)	Tangential Strength (Mpa)	Normal Fracture Energy (N/mm)	Shear Fracture Energy (N/mm)
Aggregate-mortar ITZ ^1^	3.1	9	0.03	0.09
Rubber-mortar ITZ ^2^	2.8	8.4	0.028	0.084

^1^ Data from [28], ^2^ trial values.

**Table 5:** Parameters of cement.

Type	Coagulation Time (min)		Compressive Strength (MPa)		Flexural Strength (MPa)	
	Initial Condensation	Final Condensation	3d	28d	3d	28d
P·O 42.5	180	270	26.9	50.1	5.62	8.3

**Table 6:** Parameters of aggregates.

Type	Gradation (mm)	Fineness Modulus	Apparent Density (kg/m^3^)	Stacking Density (kg/m^3^)	Water Absorption (%)	Mud Content (%)	Crushing Value (%)
Crushed stone	5–20	--	2775	1648	1.0	0.35	8.9
Sand	--	2.76	26.58	1736	1.3	1.9	--

Rubber: Crushed rubber granules from waste tyros 1–4 mm.

**Table 7:** Parameters of RC.

Type	Young’s Modulus (GPa)	Poisson’s Ratio
Mortar	36	0.2
Aggregate	72	0.16
Rubber	7	0.49

**Table 8:** Experimental and simulated peak loads.

	RC-0	RC-2.5	RC-5	RC-7.5	RC-10
Experimental peak loads (KN)	28.15	26.32	25.05	24.1	23.06
Simulated peak loads (KN)	28.62	25.94	25.52	24.44	22.23
Error (%)	1.67	−1.44	1.88	1.41	3.60

**Table 9:** Experimental and simulated fatigue life.

		RC-0	RC-2.5	RC-5	RC-7.5	RC-10
S = 0.85	experiment min/max	1615/4236	2281/3459	1485/5883	2261/6781	3827/6832
	simulation	1742	2678	3824	5018	6779
S = 0.75	experiment min/max	8654/15,432	9876/19,536	14,876/23,654	15,245/31,132	20,268/34,538
	simulation	11,812	14,208	19,081	24,085	32,742

**Table 10:** Fatigue life of different rubber doping at different stress levels.

	RC-0	RC-2.5	RC-5	RC-7.5	RC-10
S = 0.9	135	243	581	792	986
S = 0.85	1742	2678	3824	5018	6779
S = 0.8	4212	5385	9821	12036	18735
S = 0.75	11,812	14,208	19,081	24,085	32,742

**Table 11:** Fitting formulae for different rubber doping.

Type	Fitting Formula	R^2^
RC-0	$S=0.865+0.028\ln\left(N\right)-0.0043\ln^{2}\left(N\right)\;(N>26)$	0.999
RC-2.5	$S=0.671+0.087\ln\left(N\right)-0.0083\ln^{2}\left(N\right)\;(N>189)$	0.989
RC-5	$S=0.519+0.126\ln\left(N\right)-0.0104\ln^{2}\left(N\right)\;(N>361)$	0.997
RC-7.5	$S=0.475+0.135\ln\left(N\right)-0.0107\ln^{2}\left(N\right)\;(N>550)$	0.999
RC-10	$S=0.384+0.152\ln\left(N\right)-0.0112\ln^{2}\left(N\right)\;(N>886)$	0.996

## Data Availability

The data used in the article can be obtained from the author here.
